# ERN CRANIO patient coverage of craniosynostosis in Europe

**DOI:** 10.1186/s13023-022-02475-7

**Published:** 2022-09-02

**Authors:** O. Spivack, L. Gaillard, ERN CRANIO hospital representatives, Irene M. J. Mathijssen, Hans Delye, Eric Arnaud, Neil Bulstrode, David Johnson, Martin Evans, Chris Parks, Maria A. Poca, Ana Romance, Lars Kölby, Daniel Nowinski, Roberto Faggin, Carlo Giussani, Laura Valentini, Gianpiero Tamburrini, Ulrich-Wilhelm Thomale, Pia Vuola, Cláudia Faria, Federico Di Rocco, José Hinojosa Mena-Bernal, Lorenzo Genitori, Siegmar Reinert

**Affiliations:** Department of Plastic, Reconstructive and Hand Surgery, Room Ee 1591b, Dutch Craniofacial Centre, Erasmus MC – Sophia Children’s Hospital, University Medical Centre, Dr. Molewaterplein 40, 3015 GD Rotterdam, The Netherlands

**Keywords:** Craniosynostosis, Prevalence, European Reference Network, ERN, ERN CRANIO

## Abstract

**Background:**

Against the backdrop of the European Directive on patients’ rights in cross-border healthcare, 24 European Reference Networks (ERNs) were launched in 2017. ERNs are networks of specialised hospitals working together to support patients with rare and/or complex diseases. ERN CRANIO is the ERN for craniofacial anomalies and ear, nose and throat disorders. The aim of this study was to explore ERN CRANIO’s patient coverage of craniosynostosis.

**Methods:**

ERN CRANIO members and applicants were asked to retrospectively report the number of ‘new craniosynostosis patients’ (isolated and syndromic) seen in 2017. The number of live births per country in 2017 was retrieved from EUROSTAT, the EU’s statistical office. The number of new patients reported per country and the number of live births were used to generate country-specific prevalence figures per 10,000 live births. These figures were compared to expected prevalence ranges for craniosynostosis, and syndromic craniosynostosis specifically, defined by recent European studies. The percentage of syndromic craniosynostosis cases per country was also compared to the expected percentage range.

**Results:**

Based on previous studies, the expected prevalence ranges for craniosynostosis and syndromic craniosynostosis specifically were respectively defined as 4.4–7.2 and 0.9–1.6 patients/10,000 live births. For craniosynostosis (‘total’; isolated + syndromic), 'new patient' data from the UK and Finland generated prevalence figures within the expected range, and those in France, Spain, Italy, Portugal and Germany are lower than expected. However, when including applicant data, the prevalence figures for France, Spain and Italy become in range. Data from the Netherlands and Sweden generated higher prevalence figures than expected. For France, Finland, Italy and Sweden, there is inconsistency between patient coverage of ‘total’ and syndromic patients. For France, Germany, Finland and Italy, the percentage of syndromic craniosynostosis was lower than the expected range.

**Conclusion:**

ERN CRANIO’s coverage of craniosynostosis varies across Europe. Results may be explained by data collection methods, genetic testing policies and/or national healthcare systems. With centre caseload a driving force for quality, additional ERN membership calls may not necessarily ensure sufficient patient coverage for countries with decentralised healthcare systems. Liaison with national health ministries should be encouraged to optimise patient coverage.

## Background

The European Directive 2011/24 EU of the European Parliament and of the Council of 9 March 2011 on the application of patients’ rights in cross-border healthcare recognises the value of crossborder cooperation between healthcare providers providing specialist healthcare to patients with rare diseases [1]. With rare disease patient populations relatively small and scattered, clinical expertise can be sparse on a national level and information for patients, families and healthcare professionals can be limited. Against the backdrop of this European Directive 2011/24 EU, 24 European Reference Networks (ERNs) were launched in 2017 by the European Commission. ERNs are networks of healthcare professionals from healthcare providers across the European Union (EU) and European Economic Area, specialised in the care of rare and/or complex diseases [2]. ERNs seek to pool together disease-specific expertise, knowledge and resources from across Europe to ensure the provision of high-quality care to patients, regardless of where patients are located. ERN CRANIO is one of the 24 established ERNs [3]. ERN CRANIO is focused on rare and/or complex craniofacial anomalies and ear, nose and throat disorders and is coordinated by the Erasmus Medical Centre in Rotterdam, The Netherlands.

ERN member hospitals are required to meet a general and network-specific criterion [4] and membership is assessed on a disease-specific level. ERN applicants are required to submit disease-specific data on the number of patients (and new patients) that are seen and treated by their centre on an annual basis. Endorsement is also required from the relevant national ministry of health in the form of an official designation for ERN membership. With centre designation a competency of the designating country, no set limit exists on the number of hospitals per country that can be members of a given ERN. Membership applications may be submitted only after an official call for ERN membership launched by the European Commission. To date, there have been two calls for ERN membership, one in 2016 and a second in 2019. Successful applicants following the 2019 call for membership formally became part of the ERNs in January 2022.

In accordance with the ERN continuous monitoring framework, ERN members are required to provide disease-specific data on the number of new patients seen by their centre on an ongoing, annual basis. As a surgically-focused ERN, ERN CRANIO also asks centres to provide the number of ‘procedures’ performed. The new patient data collected is reported to the ERN coordinating centre for submission to the European Commission, in order to track the number of patients accessing care from ERN member hospitals over time. However, this exercise does not shed light on an ERN’s coverage of patient cases considering disease prevalence.

One of the main rare and complex disorders ERN CRANIO focuses on is craniosynostosis. Craniosynostosis is a congenital anomaly involving the premature closure of one or more cranial sutures. Craniosynostosis can occur in isolation, isolated craniosynostosis, or alongside other anomalies, syndromic craniosynostosis. A genetic cause is more likely to be identified for cases of syndromic craniosynostosis [5–7]. A diagnosis of complex craniosynostosis is provided when there is a multisutural synostosis without known genetic cause. All types of craniosynostosis fall under the scope of ERN CRANIO. The aim of this study was to explore ERN CRANIO’s patient coverage of craniosynostosis in 2017, by comparing the number of new craniosynostosis patients reported by ERN CRANIO members and successful new applicants per country to an established prevalence range.

## Methods

### Data collection

In January 2018, as part of the ERN continuous monitoring exercise, all 29 ERN CRANIO member hospitals were asked to retrospectively report their centre’s number of ‘new patients’ in 2017, for both isolated and syndromic (including complex) craniosynostosis. As part of their application, ERN CRANIO applicants following the 2nd (2019) call for ERN membership were asked to retrospectively report the same ‘new patient’ data for the past three years individually, including 2017.

The absolute numbers of ‘new craniosynostosis patients’ (for both ‘total’ [isolated + syndromic] and syndromic only—if [clearly] provided) reported by ERN CRANIO member hospitals were retrieved from internal ERN CRANIO coordination team records. Successful ERN CRANIO applicant hospitals from the same European countries as the member hospitals were identified. The absolute numbers of ‘new craniosynostosis patients’ (for both total and syndromic specifically—if [clearly] provided) reported by these successful applicants were retrieved from their online application forms.

The number of ‘new patient’ cases for craniosynostosis from ERN member hospitals, and successful applicants were calculated per country (for both total, and syndromic craniosynostosis only). The total number of live births per relevant country in 2017 was retrieved from the publicly available European Commission database provided by EUROSTAT, the statistical office of the European Union [8].

The total number of ‘new craniosynostosis patients’ per country and the number of live births per country were used to generate country-specific prevalence figures for craniosynostosis per 10,000 live births as reported by the ERN; both excluding and including the successful new applicant data. The same was done using the ‘new syndromic craniosynostosis’ patient data per country. The calculated prevalence figures based on ERN CRANIO data will be referred to as generated prevalence from this point onwards.

### Expected prevalence

The expected prevalence ranges (for total craniosynostosis, and syndromic craniosynostosis specifically) were obtained by conducting a literature search on craniosynostosis prevalence in Europe. We expected no major difference in the prevalence of craniosynostosis between European countries. The most recent studies assessing the prevalence of craniosynostosis in a European country were selected. The prevalence ranges (for total and syndromic craniosynostosis) were obtained. Multisutural craniosynostosis was included as part of syndromic craniosynostosis. The percentage of craniosynostosis patients that were reported to be syndromic was calculated for each study, and a percentage range was defined. The prevalence and percentage ranges obtained through this literature search were compared to the country-specific figures generated on the basis of 2017 ‘new patient’ data provided by ERN CRANIO members and successful new applicants, to assess ERN coverage of craniosynostosis per relevant country.

### Data analysis

Current ERN CRANIO patient coverage (for both total and syndromic craniosynostosis) was determined by investigating whether the 2017 prevalence figures generated on the basis of ‘new patient’ data from existing ERN CRANIO member hospitals were within the expected prevalence ranges established. In secondary exploratory analyses, relevant ‘new patient data’ from 2017 was included from successful hospital applications submitted in response to the 2019 call for ERN CRANIO membership. This was done to assess the impact of new ERN CRANIO members on the network’s patient coverage of craniosynostosis in those relevant countries. Consistency between ERN CRANIO’s coverage of new patients with (total) craniosynostosis and ERN CRANIO’s coverage of syndromic craniosynostosis was also assessed per country. To further assess the coverage of syndromic craniosynostosis, the percentage of new (total) craniosynostosis patients that were reported to be syndromic was also calculated per relevant country and compared to the percentage range established.

## Results

The expected prevalence ranges used in this study consider the craniosynostosis prevalence reported in two European studies conducted in Norway and the Netherlands [9, 10]. The prevalence ranges identified are shown in Table 1. The percentage of craniosynostosis cases that were reported to be syndromic (including multisutural craniosynostosis) are also displayed in Table 1. For total craniosynostosis, an expected prevalence range of 4.4–7.2 patients/10,000 live births was defined (From Norway and the Netherlands, respectively). For syndromic craniosynostosis (including multisutural craniosynostosis), an expected prevalence range of 0.9–1.6 patients/10,000 live births was defined (From Norway and the Netherlands, respectively). The percentage of craniosynostosis patients that were reported to be syndromic (including multisutural craniosynostosis) ranged from 12.3 to 36.3% (From the Netherlands and Norway, respectively).

**Table 1:** Expected prevalence of craniosynostosis

Country	Time span	Average prevalence Per 10,000 live births		First author and year
Norway	2003–2017	Total craniosynostosis	4.4	Tonne et al. [10]
		Syndromic craniosynostosis	1.6 (36.3%)	
The Netherlands	2008–2013	Total craniosynostosis	7.2	Cornelissen et al. [9]
		Syndromic craniosynostosis	0.9 (12.3%)	

Expected prevalence ranges for total and syndromic craniosynostosis, per 10,000 live births within the mentioned timeframes. These prevalence figures are based on previous country-level studies. Syndromic craniosynostosis cases include multisutural craniosynostosis. The percentage of craniosynostosis cases that were reported to be syndromic (including multisutural craniosynostosis) are also displayed

### Total craniosynostosis

**Fig. 1:**
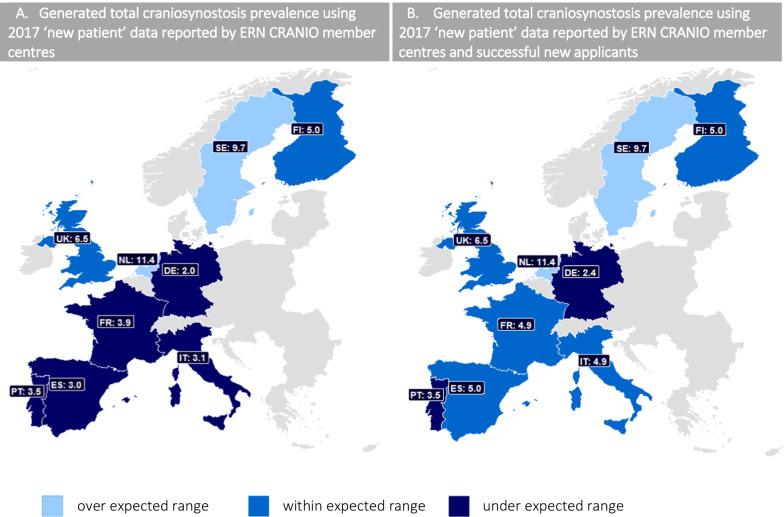
Generated total craniosynostosis prevalence using 2017 ‘new patient’ data reported by ERN CRANIO member centres and successful new applicants. **A** Displays the generated prevalence figures per 10,000 live births using data reported by ERN CRANIO member centres. No new applicant data was included. **B** Displays the generated 2017 prevalence figures per 10,000 live births using data reported by ERN CRANIO member centres and successful new applicants. Abbreviations: SE = Sweden, FI = Finland, UK = United Kingdom, NL = Netherlands, DE = Germany, FR = France, IT = Italy, ES = Spain, PT = Portugal

All ERN CRANIO member centres that reported data for (total) craniosynostosis were included in this analysis. This included 18 centres from The Netherlands, France, United Kingdom (UK), Spain, Sweden, Italy, Germany, Finland and Portugal. New patient data from one member centre in The Netherlands was excluded, as no *procedures* were reported in 2017 to ERN CRANIO. If new patients are seen at a particular centre but not surgically treated, they are likely referred on to another centre for treatment, which may result in double counting within the ERN. Four successful ERN CRANIO applicants were included from France, Spain, Italy and Germany. The absolute number of new patients reported to have craniosynostosis per country are shown in Table 2, and the numbers excluding and including the relevant ERN CRANIO applicant data are distinguished. Table 2 also displays the number of live births per country and the generated country-specific prevalence figures (per 10,000 live births), both excluding and including the successful new applicant data. Figure 1 displays an overview of the generated prevalence per participating country.

**Table 2:** Coverage of craniosynostosis. (A) Total 2017 craniosynostosis figures (per country) reported by ERN CRANIO member centres, not including successful new applicant data. (B) Total 2017 craniosynostosis figures reported by ERN CRANIO centres (per country), including successful new applicant data

Country	Number of centres	New patients	Live births 2017	Generated prevalence
*(A) Generated prevalence of total craniosynostosis*				
Higher than expected prevalence (> 7.2 per 10,000 live births)				**↑**
The Netherlands	2	193	170,000	11.4
Sweden	2	111	115,000	9.7
In expected prevalence range (4.4–7.2 per 10,000 live births)				=
Finland	1	25	50,000	5.0
UK	4	489	755,000	6.5
Under expected prevalence range (< 4.4 per 10,000 live births)				↓
Portugal	1	30	86,000	3.5
France*	1	303	770,000	3.9
Italy	4	142	458,000	3.1
Germany	1	158	785,000	2.0
Spain	2	118	391,000	3.0
*(B) Generated prevalence of total craniosynostosis including new applicant data*				
In expected prevalence range (4.4–7.2 per 10,000 live births)				=
France*	2	375	770,000	4.9
Spain	3	196	391,000	5.0
Italy	5	225	458,000	4.9
Under expected prevalence range (< 4.4 per 10,000 live births)				↓
Germany	2	185	785,000	2.4

The generated prevalence figures are provided per 10,000 live births. Number of live births was retrieved from Eurostat. *One ERN CRANIO member centre in France did not provide the number of new craniosynostosis patients and is therefore not included in these numbers.

**↑** over expected prevalence range, = within expected prevalence range, **↓** under expected prevalence range

Data from ERN CRANIO member centres in the UK and Finland generates prevalence figures that fall within the prevalence range expected. Data from ERN CRANIO member centres in France, Spain, Italy, Portugal and Germany generates prevalence figures that are lower than the expected prevalence range. However, after including patient data from new successful ERN CRANIO applicants, the prevalence figures for France, Spain and Italy become in range. The prevalence figure for Germany remains lower than the expected range, despite including successful new applicant data. Data from ERN CRANIO member centres in the Netherlands and Sweden generates prevalence figures that are higher than the expected prevalence range.

### Syndromic craniosynostosis

ERN CRANIO member centres from countries that reported complete (and clear) ‘new patient’ data for syndromic craniosynostosis specifically were included in this analysis. This included 15 centres from The Netherlands, France, UK, Sweden, Italy, Germany, Finland and Portugal. The absolute number of patients reported to have syndromic craniosynostosis per country are shown in Table 3. Table 3 also displays the number of live births per country and the generated country-specific prevalence figures (per 10,000 live births).

**Table 3:** Coverage of syndromic craniosynostosis

Country	Total no of new syndromic patients	Live births 2017	Generated prevalence	Range syndromic	Range total
Consistent with reported total craniosynostosis prevalence					
The Netherlands	44	170,000	2.6	Higher	Higher
UK	111	755,000	1.5	In range	In range
Portugal	5	86,000	0.6	Lower	Lower
Germany	18	785,000	0.2	Lower	Lower
Inconsistent with reported total craniosynostosis prevalence					
Sweden	14	115,000	1.2	In range	Higher
Finland	1	50,000	0.2	Lower	In range
Italy	17	458,000	0.4	Lower	In range
France	23	770,000	0.3	Lower	In range

Syndromic craniosynostosis figures for 2017 reported by ERN CRANIO member centres (per country). A prevalence figure is generated per 10,000 live births. Number of live births was retrieved from Eurostat. For four countries, there is inconsistency between ERN CRANIO’s patient coverage of new patients with ‘total’ and syndromic craniosynostosis, with less coverage of syndromic craniosynostosis

Absolute new patient data from ERN CRANIO member centres in the UK and Sweden generates prevalence figures that fall within the prevalence range expected. Data from ERN CRANIO member centres in France, Italy, Germany, Finland and Portugal generates prevalence figures that are lower than the expected prevalence range. Data from the Dutch ERN CRANIO member centres generates prevalence figures that are higher than the expected prevalence range.

Using the absolute new patient data, we assessed the consistency between ERN CRANIO’s patient coverage of new patients with ‘total’ and syndromic craniosynostosis. For half of the participating countries, there was inconsistency between generated prevalence for syndromic and total craniosynostosis. These countries are displayed in Table 3. Data from ERN CRANIO member centres in the Netherlands generates prevalence figures that are consistently higher than the expected prevalence ranges. Data from member centres in the UK generates prevalence figures that are consistently in range. Data from members in Germany and Portugal generates prevalence figures that are consistently lower than the expected ranges.

However, for other participating countries, there is inconsistency between ERN CRANIO’s patient coverage of new patients with ‘total’ and syndromic craniosynostosis. These countries are also displayed in Table 3. Total craniosynostosis patient data from included ERN CRANIO members and applicants in France, Finland and Italy generates prevalence figures that fall within the expected range. However, the syndromic craniosynostosis patient data from these centres generates prevalence figures that are lower than expected. For Sweden, the total craniosynostosis patient data generates a prevalence figure that is higher than the expected range but this is not reflected in the syndromic data.

**Fig. 2:**
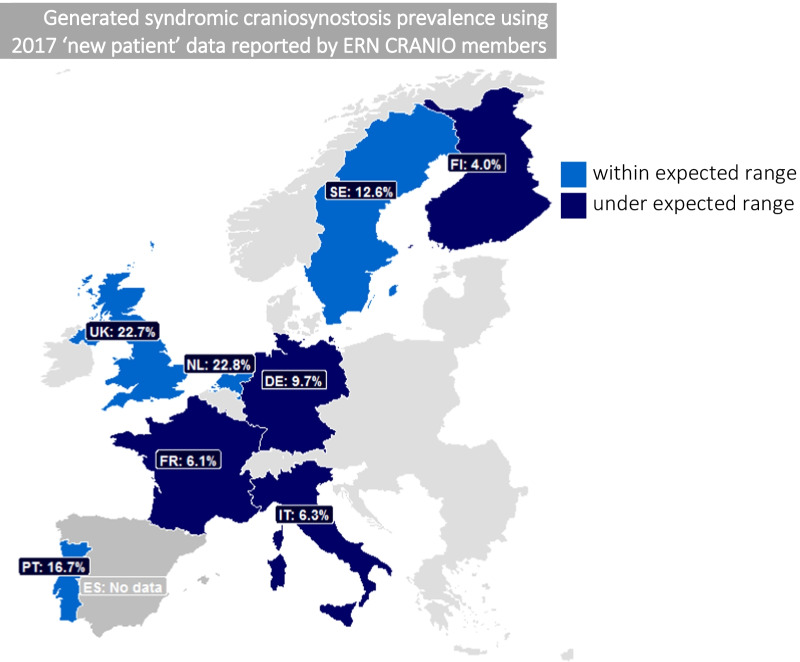
Generated syndromic craniosynostosis prevalence using 2017 new patient data reported by ERN CRANIO member centres and percentage of total craniosynostosis cases that are reported as syndromic per country. No data on syndromic craniosynostosis was available for the ERN CRANIO member centre in Spain. Abbreviations: SE = Sweden, FI = Finland, UK = United Kingdom, NL = Netherlands, DE = Germany, FR = France, IT = Italy, ES = Spain, PT = Portugal

Figure 2 and Table 4 display the coverage of syndromic craniosynostosis, considering the percentage of new (total) craniosynostosis patients that are reported to be syndromic per relevant country.

**Table 4:** Percentage of syndromic craniosynostosis

Country	Generated prevalence	Live births 2017	Percentage syndromic (%)	Range percentage syndromic
The Netherlands	2.6	170,000	22.8	In range
UK	1.5	755,000	22.7	In range
Portugal	0.6	86,000	16.7	In range
Sweden	1.2	115,000	12.6	In range
Germany	0.2	785,000	9.7	Lower
Italy	0.4	458,000	6.3	Lower
France	0.3	770,000	6.1	Lower
Finland	0.2	50,000	4.0	Lower

Percentage of new (total) craniosynostosis patients that were reported to be syndromic per relevant country

## Discussion

The aim of this study was to explore ERN CRANIO’s patient coverage of craniosynostosis in 2017, by comparing the number of new craniosynostosis patients reported by ERN CRANIO members and successful new applicants per country to an established prevalence range. The results of this study demonstrate that ERN CRANIO’s coverage of craniosynostosis varies across European countries.

New (total) craniosynostosis patient data from ERN CRANIO member centres in the UK and Finland generates prevalence figures that fall within the prevalence range expected. This implies sufficient ERN CRANIO 2017 coverage of craniosynostosis in these countries. Total craniosynostosis data from member centres in the Netherlands and Sweden generates higher prevalence figures than expected. Such higher-than-expected prevalence figures may be explained by methods of ERN CRANIO data collection. To collect retrospective 2017 data in 2018 from ERN CRANIO members, there were no explicit explanations or clarifications provided in regards to how ‘new patients’ should be defined and included. New patients of all ages and types of craniosynostosis (primary and secondary) may have been included. Also, a single patient may have been included twice if seen by two different ERN CRANIO hospitals within the time period and patients referred from a different country entirely may have also been counted. Moreover, patients with suspected, and not confirmed craniosynostosis may be included. In contrast, Cornelissen et al. [9] and Tonne et al. [10] provided stricter definitions. Cornelissen et al. [9] only included patients with diagnosed primary craniosynostosis, born in a specified time period in the Netherlands. Tonne et al. [10] similarly included diagnosed patients born in a specific time period in Norway, although they did not exclude secondary craniosynostosis explicitly. For the collection of 2018 new patient data, the ERN CRANIO coordination team made clear that only those with a confirmed diagnosis should be included. It is of note that for nine member centres included, including two centres in the Netherlands and Sweden, the number of new (total) craniosynostosis patients reported for 2018 was less than that reported for 2017.

Insufficient coverage of syndromic craniosynostosis may be in part explained by the national or local genetic testing strategy in place. A recent centre-specific study in the UK on the prevalence of genetic anomalies within a 13-year craniosynostosis birth cohort suggests that the percentage of syndromic craniosynostosis may be higher than reported by ERN CRANIO centres, as this study found 9% of patients to have a syndrome of unknown cause, 21% of patients to have clinically syndromic craniosynostosis confirmed by genetic findings and 3% of patients to be clinically non-syndromic but with genetic anomalies [6, 7]. A more intensive genetic screening strategy helps to identify genetic mutations and determine the appropriate treatment strategy [6, 7, 11], ultimately contributing to optimised healthcare for patients with craniosynostosis. In line with this, the recently updated Dutch guideline for craniosynostosis, which is endorsed by ERN CRANIO, recommends genetic diagnostics for patients with confirmed craniosynostosis [11]. With there being no national routine genetic testing protocol in place at the time of the Cornelissen study [9], the lower limit of the prevalence range used in this study for syndromic craniosynostosis may also be underestimated.

In addition, it should be noted that data collection for syndromic craniosynostosis was suboptimal. ERN CRANIO applicants were excluded from the syndromic analyses outlined in this paper due to incomplete and/or unclear data. The ERN CRANIO syndromic craniosynostosis prevalence figures for France, Germany and Italy are therefore likely underestimated. Additionally, one ERN CRANIO member centre (Spain) was excluded due to not reporting on syndromic craniosynostosis specifically, and therefore no prevalence figure for ‘syndromic craniosynostosis’ could be generated for Spain.

An ERN-wide definition of ‘new patient’ and additional clarifications for data collection have since been documented. ‘New patient’ is currently defined as; *“The total number of new patients attending the ERNs’ Health Care Provider for the first time during the reporting period, whose disease or condition falls within the scope of the ERN, whatever their age, including visits to outpatient clinics, hospital discharges and emergencies, coming from national and international referrals.”* Patients are to be included only if they have a confirmed diagnosis and if they have not previously been included in the patient information system of the healthcare provider. However, despite such clarifications in place, collecting healthcare providers’ data in this way is still not optimal for generating reliable disease prevalence figures or estimating ERN patient coverage considering disease prevalence. An ERN CRANIO patient registry is currently in development. Registering each patient that visits an ERN CRANIO centre will help to improve the reliability and accuracy of ERN CRANIO data collection for this purpose. Double inclusion will be prevented, and established diagnosis, date of birth and country of residence will be recorded, allowing ERN CRANIO to improve patient selection further. Additionally, genetic information will be recorded, which will allow for a more accurate assessment of the prevalence and coverage of syndromic and isolated craniosynostosis. For disorders like craniosynostosis requiring surgical intervention, the number and type of surgical procedures are also important to include in the ERN CRANIO registry in order to monitor centre experience and expertise. In the meantime, clear, accurate definitions and instructions should be given to ERN CRANIO centres to improve data collection and all reported syndromic craniosynostosis cases should have genetic confirmation.

On a national level, the provision of healthcare to patients with rare and/or complex diseases varies, with some healthcare systems operating regionally and others centrally. Countries known to have centralised healthcare systems in place for craniosynostosis include the Netherlands, Finland, Sweden and the UK. All centres included from these countries are existing ERN CRANIO member centres and there is sufficient ERN CRANIO patient coverage for (total) craniosynostosis based on the expected prevalence range. For other countries (France, Italy, Spain) the generated prevalence figures fall within the expected prevalence range with the inclusion of successful new applicant data. However, the countries differ in regards to the number of centres contributing data. For Italy, a country with a healthcare system operating regionally, data is provided by five centres and new patient numbers for (total) craniosynostosis range between 22 and 83 per centre. The centralisation of care for rare diseases can be a politically sensitive topic in European countries with decentralised healthcare systems. However, ERN membership is in part determined by the number of patients a centre sees for the first time and the number of patients it treats annually; a centre’s caseload is considered a driving force for quality. Therefore, additional calls for ERN membership may not necessarily ensure sufficient ERN patient coverage for countries with decentralised healthcare systems.

Our study has some limitations. Firstly, it was assumed that the prevalence of craniosynostosis is similar across Europe. Although we do not expect large differences across Europe, there have been no previous studies in several of the participating countries to confirm this. Additionally, as previously noted, the definitions used to include patients in the previous prevalence studies [9, 10] may have differed from those used to report ERN CRANIO centre and applicant data. Such retrospective data collection also relies on accurate data storage and extraction, which may be manually completed in some cases and run the risk of error. There may also be local and national variation. To improve data quality, data validation methods should be incorporated into the ERN data collection process. Extraction method monitoring and local funding to support data extraction may also help to improve data quality [12].

## Conclusion

ERN CRANIO’s coverage of craniosynostosis patients varies across Europe. Results may partly be explained by methods of data collection. ERN data collection should be optimised for the purpose of generating reliable disease prevalence figures or estimating ERN patient coverage considering disease prevalence and genetic testing is important to ensure accurate detection of syndromic craniosynostosis. A centre’s surgical activity should also be monitored as a key marker of expertise in craniosynostosis care. Cross-country differences in ERN CRANIO coverage may also be explained by the national healthcare system in place. With a centre’s caseload considered a driving force for quality within the ERNs, additional calls for ERN membership may not necessarily ensure sufficient ERN patient coverage for countries with decentralised healthcare systems. ERN CRANIO aims to provide equal optimised care for patients with craniosynostosis across Europe by pooling together expertise on this rare disorder. Ensuring adequate ERN CRANIO coverage across all European countries is key to reaching this goal. With centre designation a competency of the individual European country, this study can be used as a reference for discussion with local health ministries to promote optimal ERN CRANIO disease coverage in the future.

## Data Availability

All data generated or analysed during this study are included in this published article.
